# Implementation and effects of Movement-oriented Restorative Care in a nursing home – a quasi-experimental study

**DOI:** 10.1186/s12877-017-0642-x

**Published:** 2017-10-23

**Authors:** Marinda Henskens, Ilse M. Nauta, Erik J. A. Scherder, Frits G. J. Oosterveld, Susan Vrijkotte

**Affiliations:** 1 Department of Clinical Neuropsychology, VU University Amsterdam, van der Boechorststraat 1, 1081 BT Amsterdam, Netherlands; 2 Department of Neurology, VU University Medical Center, de Boelelaan 1117, 1081 HV Amsterdam, Netherlands; 3 Saxion University of Applied Science, P.O. Box 75.000, 7500 KB Enschede, Netherlands; 4 Zorggroep Solis, Postbus 5014, 7400 GC Deventer, Netherlands; 5 Human Physiology Research Group, VU University Brussel, Pleinlaan 2, B-1050 Brussels, Belgium

**Keywords:** Dementia, Movement, Quality of life, Activities of daily living, Institutionalization

## Abstract

**Background:**

The prevalence of dementia is expected to increase rapidly, and institutionalization is a common consequence of the disease. Dependence in activities of daily living (ADL) is a predictor for institutionalization and a determinant for the quality of life (QoL). A promising method to increase functional independence in nursing homes is a restorative care or function focused care (FFC) approach. Movement-oriented restorative care (MRC) is derived from the concept of FFC and restorative care and focuses on the integration of physical activity in the daily lives of nursing home residents with dementia using a multidisciplinary approach. The objective of this study was to assess the effectiveness of MRC in preservation of ADL independence and QoL in nursing home residents with dementia.

**Methods:**

In this quasi-experimental 12-month study, the effects of MRC were compared to care as usual in 61 nursing home residents with moderate to severe dementia. The outcome measures were ADL independence and QoL. These outcomes were measured five times (i.e. at baseline, and after 3, 6, 9, and 12 months). Additionally, data was collected regarding the degree of implementation, and the barriers to the implementation process. The effect of the intervention was analyzed using linear mixed model analyses.

**Results:**

There was no significant overall intervention effect on ADL independence and QoL. A significant group-by-time interaction was found for the QoL subscale positive self-image: after a 12 month intervention period, the MRC group scored significantly better than the control group on positive self-image. Regarding the other subscales and the total score of the QoL, as well as ADL, no significant group-by-time interactions were found.

**Conclusions:**

MRC did not demonstrate significant improvements in ADL or QoL. After a 12-month intervention period, residents who received MRC showed higher scores on positive self-image compared to the control group. This study contributes to the limited research regarding the effect of MRC on resident outcomes. Further large-scale studies are recommended.

**Trial registration:**

The trial was retrospectively registered in http://clinicaltrials.gov on February 2, 2017: NCT03001232.

## Background

Worldwide, 47.5 million people are diagnosed with dementia [1]. Due to the aging population and the absence of a cure for dementia, this number is expected to increase rapidly in the coming years [1]. In the Netherlands, an estimated number of 70,000 patients with dementia are currently institutionalized [2]. An important predictor for institutionalization is a loss of independence in activities of daily living (ADL) [3], and independence in ADL is a key determinant for the quality of life (QoL) of patients with dementia [4]. Therefore, it is important to attain the highest possible level of functional independence in ADL.

However, once institutionalized, independent functioning tends to decline more rapidly than expected based on the neuropathology [5]. The study of Carpenter and colleagues (2006) shows that 6 months after institutionalization, residents with moderate cognitive impairment show a decline in ADL [6]. This rapid decline may be due to insufficient stimulation of the patients’ remaining abilities, as nursing staff may overestimate physical and cognitive limitations of patients with dementia [7, 8]. In addition, adequate assistance of patients with dementia during ADL may be a challenge due to frequently occurring behavioral and mood problems [5, 9]. Furthermore, the inactive lifestyle often observed among nursing home (NH) residents [10] may also contribute to the rapid decline in ADL [11].

A promising method described in the literature to increase functional independence in ADL in NH residents with dementia is a restorative care or function focused care (FFC) approach, terms which are interchangeably used to describe a philosophy of care that focusses on obtaining the highest level of functional independence by stimulating physical activity during performance of ADL throughout the day [12]. Since most basic ADLs, such as bathing and toileting, are overlearned behaviors, it is argued that these functions can still be trained in patients with dementia [13, 14]. Studies implementing FFC, restorative care, or other comparable interventions reported inconsistent results. Some studies indicate positive effects on ADL independence [5, 12, 15–17], while others found no beneficial effects [18–21]. To our knowledge, no improvements are found for QoL measures [12]. In general, studies do indicate that the nursing staff is willing to actively stimulate NH residents to increase physical activity [5, 12, 16].

Since only a small number of these studies focused on NH residents with moderate to severe dementia [12], it is relevant to further investigate physical stimulation in this patient group. It is possible that in comparison to cognitively healthy elderly, NH residents with dementia need a long intervention period for changes to be effectuated [20]. The majority of the studies described, however, had a relatively short intervention period [12]. Moreover, adopting a multidisciplinary approach when stimulating physical activity could lead to more continuous stimulation of physical activity in all aspects of care throughout the day. The approach should therefore integrate various disciplines in order to increase functional independence in ADL.

Therefore, the purpose of this study was to test the effect of movement-oriented restorative care (MRC) among NH residents with moderate to severe dementia. MRC is derived from the concept of FCC and restorative care [12] and focuses on the integration of physical activity in the daily lives of NH residents with dementia using a multidisciplinary approach. MRC incorporates a wide range of disciplines, including nursing staff, department heads, physiotherapists, occupational therapists, psychologists, geriatricians, and activity leaders, as well as volunteers and family members. It is an individualized approach based on cognitive and physical capabilities of the resident, taking into account individual preferences and motivation. The goal of MRC is to optimize independence in ADL and QoL by continuously stimulating physical activity in ADL throughout the day. It was hypothesized that a 12-month MRC approach results in a maintenance, or a slower decline of ADL independence and QoL in NH residents with dementia compared to residents with dementia who receive care as usual.

## Methods

### Design

A 12-month quasi-experimental study with two parallel groups and a convenient sample was conducted. Two locations of a long-term care organization (‘Solis’) in the Netherlands were non-randomly assigned to the intervention or control location, due to ethical and practical considerations. The study was approved by a local institutional review board and the medical ethical review committee of the VUmc. Written consent was obtained from the legal representatives of the participants.

### Participants

**Fig. 1:**
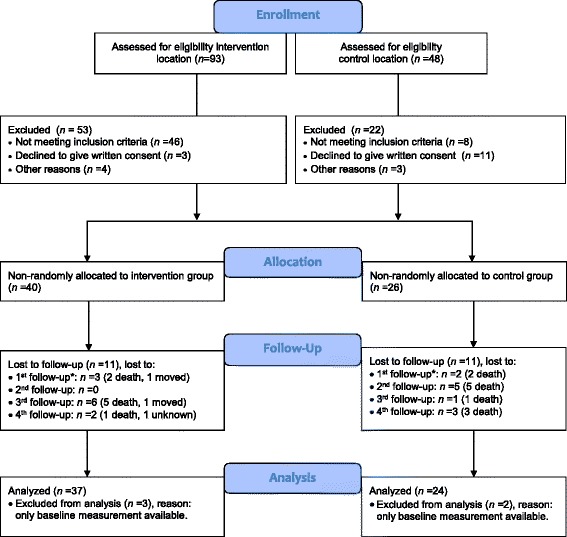
Flow diagram of the sampling procedure. Note. *Excluded from analysis (only baseline data)

In total, there were 93 residents living in the psychogeriatric ward of the intervention location, and 48 living in the control location. Inclusion criteria were (1) diagnosis of dementia, (2) living in a psychogeriatric ward of a Dutch nursing home for at least 3 weeks, and (3) 65 years of age or older. Exclusion criteria were (1) very bad vision, (2) psychotic symptoms, (3) very severe dementia (those who receive PDL care; care of people who are Powerless in Daily Living) [22], (4) a score on the Mini-Mental State Examination (MMSE) [23] of 24 or higher, and (5) medical contraindications for participating in physical activities. In total, 66 participants were included in this study, of which 5 participants were excluded from analysis due to drop-out before the first follow-up. Of the 61 participants analyzed, 37 participants were allocated to the intervention condition and 24 participants to the control condition. Of these participants, 29 and 15 participants completed the study respectively. The drop-out rate did not differ significantly between the intervention group (*n* = 8, 21.6%) and the control group (*n* = 9, 37.5%), *χ* ^2^ = 1.83, *p* = .244. A flow-diagram of the sampling procedure and the reasons for drop-out are represented in Fig. 1.

#### Demographics

Factors considered were age, gender, type of dementia, and severity of cognitive impairment. Type of dementia was derived from the residents’ medical records. The severity of cognitive impairment was determined by the scores on the MMSE [23]. The scores ranged from 0 (severe cognitive impairment) to 30 (no cognitive impairment). The recommended cut-off point of 24 was applied, with a score of 23 or lower indicating dementia [23].

### Materials and procedure

#### Materials

##### Activities of daily living (ADL)

The Barthel Index is designed to measure the level of independence in ADL [24]. The survey consists of 10 performance items (e.g. toilet use, dressing) and each item can be rated on a two, three- or four-point scale indicating the level of help needed. The survey is completed by two professional caregivers; the first responsible caregiver and a qualified nursing staff. Scores range from 0 to 20, with higher scores representing a higher level of functional independence. The Barthel Index is considered a reliable and valid measure of functional disability [25, 26].

##### Quality of life (QoL)

QoL was assessed with the Qualidem [27, 28]. The questionnaire includes 37 items for elderly with mild-to-severe dementia [27]. The items measure observable behavior and each question is rated on a four-point scale, ranging from 0 (never) to 3 (often). The questionnaire consists of nine subscales: (1) care relationship (range: 0–21), (2) positive affect (range: 0–18), (3) negative affect (range: 0–9), (4) restless tense behavior (range: 0–9), (5) positive self-image (range: 0–9), (6) social relations (range: 0–18), (7) social isolation (range: 0–9), (8) feeling at home (range: 0–12), and (9) having something to do (range: 0–6). For each subscale, a higher score represented a better QoL. The mean total Qualidem score was calculated by adding the mean score of each of the 9 subscales (range: 0–27). The questionnaire is completed by the first responsible caregiver together with a qualified nursing staff. The scales are moderately reliable [27, 29] and valid for elderly with dementia in a residential setting [28].

### Procedure

Residents were non-randomly allocated to the intervention or control condition, based on their living location, due to practical and ethical considerations. Moving residents to another ward was considered too much of a burden for the resident. Additionally, by separating the control and intervention location, staff from the intervention location were prevented from sharing their knowledge with staff from the control location. The intervention location consisted of three psychogeriatric wards and the control location of two. The psychogeriatric wards were large-scale homes with living rooms with a maximum capacity of 30 residents. Participants living at the intervention location received MRC, while participants living at the control location received care as usual. The outcome variables were measured five times, i.e. at baseline and 3, 6, 9, and 12 months after baseline. Whenever possible, the participants were rated by the same caregiver throughout the study.

#### Movement-oriented Restorative Care (MRC)

MRC is derived from the concept of FCC and restorative care [12], which is a multidisciplinary approach towards nursing home dementia care that focuses on stimulating physical activity and independent functioning in daily care and activities throughout the day. MRC incorporates important aspects of FFC and restorative care, such as establishing goals with each resident, educating nursing staff and families, and administering process evaluations to determine the extent to which MRC was implemented as intended [20]. In addition to these elements, MRC incorporates a broad range of disciplines, such as nursing staff, department heads, physiotherapists, occupational therapists, psychologists, geriatricians and activity supervisors. Additionally, volunteers and family caregivers are involved in MRC. Each discipline had a different role in MRC. First, the primary responsible caregiver played an important role in the initial selection and evaluation of the functional capabilities of each resident, as they are most involved in the residents’ life, and therefore familiar with their capabilities, history, and preferences. Consequently, the geriatricians advised the nursing staff about the specific medical and physical capabilities and limitations of each participant, ensuring safe physical stimulation. Based on the final advice of the geriatrician, the nursing staff created a plan for each participant to determine which daily routines and activities could be performed independently, and in what manner they could be stimulated to engage in physical activities. Individually based instructions were reported in the following four domains of the health care plan: (1) living conditions, (2) participation, (3) mental well-being and autonomy, and (4) physical well-being and health. In addition, activity supervisors constructed a leisure-care calendar that focused on general activities based on physical capabilities and personal preferences. The nursing staff played a central role in MRC by continuously stimulating participants to function as independently as possible. Family caregivers and volunteers were encouraged to stimulate the participants in being physically active in general. Finally, the heads of departments ensured MRC was discussed during staff meetings.

#### Training and information

Nursing staff were intensively trained by an expert in MRC (three sessions of 3 h). The MRC expert is a professional and qualified trainer in the area of increased self-control and functional independence amongst elderly, directed at stimulating change in the geriatric sector. The trainer aimed to increase awareness of the benefits of physical activity throughout the day, the role of physical activity in ADL, the stimulating and motivational role of the nursing staff, how physical activity can be integrated into their daily work routine, and how to document physical activity in the life care plan. To translate this knowledge into practice, the trainer focused on practical techniques to provoke independence in ADL, and to stimulate physical activity throughout the day. Additionally, physiotherapists and occupational therapists were informed in a two-hour meeting about the benefits of physical activity and their role in advising the nursing staff about stimulation of physical activities in ADL. Furthermore, the volunteers and family caregivers were informed about the benefits of an active lifestyle and the role of physical activity in ADL during two open meetings, where they also had the opportunity to ask questions. The open meetings were given by a qualified staff member who was previously trained by the MRC expert. Additional workshops were given in which family caregivers and volunteers could experience the benefit of movement, as well as receive practical tips on how to stimulate resident activity. Lastly, they received written information at home.

#### Compliance to the intervention

To ensure compliance to the intervention, one qualified staff member at each psychogeriatric ward became an ‘ambassador’ of MRC. Ambassadors were responsible for warranting the continuation of MRC at their ward. Compliance to the intervention was determined by administering process evaluations, which included both questionnaires and structured interviews. The structured interviews integrated five theoretical elements from previous studies [30–32] (see Table 1). Each element consisted of several corresponding questions which could be answered on a 5- or 10-point Likert scale. The average of these scores were reported. Structured interviews were conducted with members of a multidisciplinary focus group after three and 9 months, and evaluated the intervention on a group level per psychogeriatric ward. The focus group consisted of the following 12 professionals: three nurses, three activity supervisors, two heads of the departments, one physiotherapist, one occupational therapist, and two ‘ambassadors’. Questionnaires were completed by the nursing staff and family caregivers every 3 months, and reflected individual patient based evaluations of the intervention.

**Table 1:** Theoretical elements and measuring method of process evaluations

Theoretical element	Research Method	Measuring Method	
		Questionnaires	Focus group
Dose delivered	Which aspects of MRC are applied?	x	x
Fidelity	Is MRC applied according to its core principles?	–	x
Exposure	To what extent are participants stimulated to be physically active?	–	x
Satisfaction	Are the staff and family carers satisfied with (the execution of) MRC?	x	x
Barriers	What barriers are experienced?	–	x

x = theoretical element is included in the measuring method, − = theoretical element not included

### Statistical analysis

Statistical analyses were performed using IBM Statistical Package for the Social Sciences (SPSS) 23.0. Differences between groups in demographic characteristics and outcome measurements at baseline were analyzed using independent samples t-tests, Mann-Whitney U tests, and Pearson’s chi-square tests.

The effect of the intervention was analyzed according to a modified intention-to-treat analysis, including participants with at least one follow-up measurement, using linear mixed model (LMM) analyses. Two hierarchal levels were distinguished. Data was analyzed using two models. The first model examined the overall effect of the intervention, independent of time. The second model examined the effect of the intervention at each time point. Group, time and the group-by-time interaction were inserted as predictors to the model. Random intercepts and slopes at participant-level were included if significantly improving model fit using the Likelihood Ratio Test. Data were analyzed using a crude and an adjusted model. In the crude model, baseline performance was added as a covariate. In the adjusted model, baseline performance, gender, age, and MMSE were added as covariates to the model. For the final models, a dichotomous ‘completed’ group variable was added as a covariate, representing completion of the study or lost between the first and last measurement, to examine whether drop-outs influenced the intervention effect. Last, it was examined whether the residuals were normally distributed in the final models.

Alpha level was set at .05 for baseline characteristics and the outcome variable ADL. For the nine quality of life subscales and its total score, a Bonferroni correction was used to correct for alpha inflation (*p* < .005 (.05/10)). Regression coefficients and 95% confidence intervals for both models were reported, with the regression coefficients representing the overall intervention effect for model 1 (group estimate) and the difference of the intervention effect at different time points for model 2 (group by time estimate).

## Results

### Comparisons at baseline

The demographics of the group of participants included in the analyses are listed in Table 2. The average MMSE scores indicate a severe stage of dementia in both the intervention and control group. However, on average, the intervention group scored significantly higher than the control group on MMSE at baseline (score 9.75 *v* 6.52; *p* < .05). The participants in the intervention group did not differ significantly from the participants in the control group on baseline ADL and QoL (all *p’s* > .05, see Table 3). The 17 participants who dropped out between the first and last follow-up measurement did not differ significantly from the 44 participants who completed the study on demographic characteristics (all *p’s* > .05). The participants who dropped out did have significantly lower scores at baseline on the Barthel Index (*p* < .005), and the subscales ‘social relations’ (*p* < .005) and ‘having something to do’ of the Qualidem (*p* < .05).

**Table 2:** Demographic characteristics of the participants at baseline

	Intervention group (*n* = 37)	Control group (*n* = 24)
Age, mean *(SD)*	86.51 (7.1)	84.21 (4.7)
Age, range	69–100	70–92
Gender (female), *n* (%)	30 (81.1)	17 (70.8)
MMSE, mean (SD)*	9.75 (5.1)	6.52 (5.2)
Diagnosis, *n* (%)*		
Alzheimer’s disease	21 (56.8)	13 (54.2)
Vascular dementia	2 (5.4)	5 (20.8)
Mixed vascular and Alzheimer	1 (2.7)	5 (20.8)
Other/unknown	13 (35.1)	1 (4.2)

*MMSE* Mini-Mental State Examination, *rf* risk factor

*p < .05

**Table 3:** Means of ADL and QoL ratings at each measurement of participants^a^

	Group	T0 *N* = 61 *n*_int_ = 37 *n*_con_ = 24 *M(SD)*	T1 N = 61 *n*_int_ = 37 *n*_con_ = 24 *M(SD)*	T2 *N* = 55 *n*_int_ = 36 *n*_con_ = 19 *M(SD)*	T3 *N* = 49 *n*_int_ = 31 *n*_con_ = 18 *M(SD)*	T4 *N* = 44 *n*_int_ = 29 *n*_con_ = 15 *M(SD)*
Barthel index	Int.	9.38(5.37)	9.16(4.76)	8.72(4.96)	8.55(5.57)	8.86(5.28)
	Con.	8.50(5.79)	7.54(5.28)	8.89(5.43)	8.17(5.84)	8.27(5.65)
Qualidem						
care relationship	Int.	14.46(3.98)	14.27(4.33)	14.58(4.00)	14.61(3.84)	14.86(4.21)
	Con.	12.58(4.01)	13.25(3.85)	12.32(3.48)	12.50(4.38)	11.67(3.66)
positive affect	Int.	14.51(3.66)	14.16(3.89)	14.17(3.08)	14.61(3.78)	14.21(3.42)
	Con.	13.63(3.19)	13.96(2.71)	14.32(2.96)	13.39(3.18)	14.93(2.25)
negative affect	Int.	5.43(2.49)	5.38(2.70)	5.25(2.53)	5.90(2.45)	5.55(2.38)
	Con.	5.79(2.62)	5.54(2.15)	5.32(2.31)	5.06(2.90)	4.47(2.33)
restless tense behavior	Int.	4.27(2.81)	4.08(2.87)	4.19(2.84)	4.42(3.05)	4.52(2.95)
	Con.	4.21(2.28)	4.17(2.35)	4.47(2.78)	4.00(2.68)	4.07(2.66)
positive self-image	Int.	5.89(2.48)	5.59(2.82)	5.72(2.72)	6.71(2.37)	6.07(2.74)
	Con.	6.88(2.07)	7.08(1.53)	6.68(1.42)	6.78(2.16)	4.33(2.38)
social relations	Int.	11.73(4.05)	11.62(3.78)	11.92(3.89)	12.06(4.29)	11.97(3.91)
	Con.	10.46(4.30)	10.00(4.56)	10.95(3.94)	10.28(4.24)	10.87(3.89)
social isolation	Int.	5.62(2.37)	5.32(2.47)	5.53(2.22)	5.94(2.35)	6.00(2.49)
	Con.	5.38(2.14)	5.21(1.87)	5.21(1.87)	4.83(2.20)	4.60(1.92)
feeling at home	Int.	9.24(2.64)	9.68(2.52)	9.50(2.31)	10.06(1.91)	10.10(2.68)
	Con.	8.54(2.67)	8.88(2.77)	7.84(2.85)	8.17(3.42)	7.93(3.47)
having something to do	Int.	2.24 (1.91)	2.62(1.93)	2.25(1.92)	2.29(1.85)	2.66(1.97)
	Con.	1.92(1.56)	1.79(1.25)	2.11(1.79)	2.22(1.56)	2.53(1.46)
mean total QoL	Int.	16.94(4.09)	16.86(5.08)	16.83 (4.71)	17.85 (4.81)	17.72 (4.95)
	Con.	16.32(3.47)	16.33(2.32)	16.21 (1.93)	15.77 (2.75)	15.03 (3.08)

*Int* Intervention group, *Con* Control group, T0 = baseline; T1 = 3 months; T2 = 6 months; T3 = 9 months; T4 = 12 months

^a^High rating indicates better independence in ADL and better QoL

### Intervention effect

The parameter estimates of the final crude and adjusted multilevel models are presented in Table 4. There was no significant main intervention effect on the Barthel Index (*p* = .622) and the subscales and total score of the Qualidem (all *p’s* > .005). This holds for both the crude and adjusted analyses.

**Table 4:** Overall intervention effect on ADL and QoL unadjusted and adjusted for confounders

Intervention vs. control	Crude Model		Adjusted Model	
	Beta(95% CI)	*p*-value	Beta(95% CI)	*p*-value
Barthel index	0.62(−0.42;1.67)	0.24	0.27(−0.83;1.37)	0.62
Qualidem				
care relationship	0.71(−0.72;2.15)	0.32	0.69(−0.85;2.23)	0.37
positive affect	−0.15(−1.38;1.08)	0.81	−0.44(1.74;0.85)	0.50
negative affect	0.48(−0.28;1.24)	0.21	0.56(−0.28;1.39)	0.19
restless tense behaviour	0.23(−0.80;1.26)	0.66	0.02(−1.07;1.11)	0.97
positive self-image	0.15(−0.72;1.01)	0.74	0.28(−0.67;1.24)	0.55
social relations	1.12(−0.19;2.43)	0.09	1.01(−0.37;2.38)	0.15
social isolation	0.47(−0.29;1.23)	0.22	0.72(−0.04;1.47)	0.06
feeling at home	0.94(−0.04;1.93)	0.06	0.84(−0.25;1.94)	0.13
having something to do	0.17(−0.44;0.77)	0.58	0.09(−0.55;0.74)	0.78
mean total QoL	0.96(−0.52;2.43)	0.20	0.95(−0.65;2.56)	0.24

Crude model: adjusted for baseline scores; Adjusted model: adjusted for baseline scores, age, gender and MMSE

Table 5 shows the intervention effect at different time points. There was no significant group-by-time interaction effect for the Barthel Index, indicating that intervention effects did not differ over time between the MRC and control group. With respect to the Qualidem, a significant group-by-time interaction was found for the subscale positive self-image. Specifically, the MRC group scored significantly better than the control group on the subscale positive self-image after 12 months (*b =* 2.36, *p* < .001 in the adjusted analysis). Regarding the other subscales and the total score of the Qualidem, no significant group-by-time interactions were found after correcting for alpha inflation (*p* > .005, see Table 5). The dummy variable ‘completion’ (study completed versus lost to follow-up) was a significant predictor of the Qualidem subscale ‘feeling at home’ for males, yet, it did not influence the group-by-time estimate. Due to the adjustment for baseline values, all models had normally distributed residuals.

**Table 5:** Intervention effect at different time-points on ADL and QoL unadjusted and adjusted for confounders

Intervention vs. Control		T1		T2		T3		T4	
	Model	B(95% CI)	*p*	B(95% CI)	*p*	B(95% CI)	*p*	B(95% CI)	*p*
Barthel Index	Cru.	0.88(−0.38;2.14)	0.17	0.05(−1.28.;1.38)	0.94	0.49(−0.88;1.87)	0.48	1.32(−0.12;2.76)	0.07
	Adj.	0.30(−0.99;1.60)	0.81	−0.29(−1.65;1.07)	0.67	0.29(−1.11;1.68)	0.69	1.31(−0.15;2.78)	0.08
Qualidem									
Care relationship	Cru.	−0.13(−1.83;1.57)	0.88	1.16(−0.64;2.95)	0.21	1.08(−0.77;2.93)	0.25	1.32(−0.62;3.26)	0.18
	Adj.	0.17(−1.60;1.94)	0.85	1.11(−0.73;2.96)	0.24	0.64(−1.26;2.53)	0.51	1.12(−0.87;3.10)	0.27
Positive affect	Cru.	−0.24(−1.70;1.21)	0.74	−0.26(−1.79;1.28)	0.74	0.89(−0.70;2.47)	0.27	−1.09(−2.75;0.56)	0.19
	Adj.	−0.45(−1.97;1.07)	0.56	−0.64(−2.23;0.95)	0.43	0.53(−1.11;2.17)	0.52	−1.40(−3.12;0.31)	0.11
Negative affect	Cru.	0.06(−0.91;1.03)	0.90	0.11(−0.91;1.14)	0.83	1.18(−0.12;2.24)	0.03	1.00(−0.11;2.12)	0.08
	Adj.	0.02(−1.01;1.05)	0.97	0.27(−0.81;1.36)	0.62	1.34(−0.22;2.46)	0.02	1.08(−0.11;2.26)	0.72
Restless tense- behavior	Cru.	−0.12(−1.32;1.07)	0.84	0.03(−1.23;1.29)	0.96	0.77(−0.53;2.06)	0.24	0.48(−0.87;1.82)	0.48
	Adj.	−0.41(−1.68;0.85)	0.52	−0.06(−1.38;1.26)	0.93	0.58(−0.76;1.93)	0.40	0.25(−1.16;1.66)	0.73
Positive self-image	Cru.	−0.91(−1.95;0.13)	0.09	−0.36(−1.46;0.75)	0.52	0.59(−0.55;1.73)	0.31	2.35(1.15;3.55)	**0.00**
	Adj.	−0.71(−1.83;0.41)	0.21	−0.29(−1.47;0.89)	0.63	0.85(−0.37;2.07)	0.17	2.36(1.09;3.64)	**0.00**
Social relations	Cru.	0.71(−0.77;2.20)	0.34	1.02(−0.53;2.57)	0.19	1.67(0.08;3.25)	0.04	1.39(−0.25;3.03)	0.10
	Adj.	0.67(−0.88;2.22)	0.40	0.97(−0.64;2.58)	0.24	1.45(−0.21;3.10)	0.08	1.21(−0.51;2.91)	0.17
Social isolation	Cru.	−0.03(−0.96;0.91)	0.96	0.27(−0.73;1.26)	0.60	1.02(−0.01;2.05)	0.05	0.94(−0.14;2.03)	0.09
	Adj.	0.31(−0.63;1.26)	0.51	0.51(−0.49;1.51)	0.31	1.21(−0.17;2.24)	0.02	1.09(−0.00;2.18)	0.05
Feeling at home	Cru.	0.44(−0.73;1.62)	0.46	1.09(−0.16;2.34)	0.09	1.40(0.12;2.69)	0.03	1.21(−0.13;2.56)	0.08
	Adj.	0.35(−0.92;1.63)	0.58	0.97(−0.38;2.31)	0.16	1.34(−0.77;1.92)	0.39	1.05(−0.40;2.51)	0.15
Having something to do	Cru.	0.63(−0.10;1.36)	0.09	0.06(−0.71;0.83)	0.87	−0.23(−1.03;0.56)	0.56	−0.06(−0.89;0.77)	0.89
	Adj.	0.51(−0.26;1.27)	0.19	0.00(−0.80;0.81)	0.99	−0.25(−1.07;0.58)	0.56	−0.14(−1.01;0.73)	0.75
Mean total QoL	Cru.	0.11 (−1.55;1.77)	0.90	0.58 (−1.16;2.32)	0.51	1.96 (0.18;3.74)	0.03	2.04 (0.20;3.88)	0.03
	Adj.	0.14 (−1.65;1.93)	0.88	1.39 (−0.61;3.39)	0.17	1.96 (0.05;3.86)	0.04	1.89 (−0.09;3.86)	0.06

Bold values indicate significant *p-*values after Bonferroni correction

*Cru* crude model, *Adj* adjusted model; T1 = effect after 3 months; T2 = effect after 6 months; T3 = effect after 9 months; T4 = effect after 12 months

### Compliance to the intervention

#### Process evaluations

A summary of the process evaluations is presented in Table 6.

**Table 6:** Summary of process evaluations regarding compliance to the intervention

Theoretical element	Nursing Staff^a^	Family Caregivers^a^	Focus group^b^
Dose delivered (scale 1 to 5)ª	3.54	–	3.21
Fidelity (scale 1 to 5)ª	–	–	3.24
Exposure (scale 1 to 5)ª	–	–	3.27
Satisfaction (scale 1 to 10)^b^	6.86	6.45	6.88
Experienced benefits of MRC (%)	78.6	22.2	–
Willing to continue with MRC (%)	92.90	89.50	–

ª1 = very bad, 5 = very good, ^b^1 = very bad, 10 = very good, ^c^questionnaires, ^d^interviews

##### Reach

All 37 participants of the intervention group were reached. In addition, all permanent staff members of the psychogeriatric ward were trained in MRC. The nursing staff analyzed each individuals’ physical potential and reported individually based instructions for each domain in the health care plan.

##### Dose delivered

The program was individually based, and therefore, there was no standard protocol. In Table 7, an example of an individually based health care plan is presented.

The amount of stimulation varied from three times per week to several times per day. The nursing staff rated their preparedness to deliver MRC a 4.06 on a scale from 1 (very bad) to 5 (very good).

**Table 7:** Example of an individually based health care plan

Domain	Example from a participant who could walk independently
1.Living conditions	Client tidies up her apartment herself and puts the laundry onto her bed. The cleaning is done by the service agency. Client can independently prepare breakfast and can take the warm food from the pan onto the plate.
2. Participation	Client likes to go to almost all activities and likes to be busy. Client likes to go outside. Client can be stimulated to activities and physical activity when offered in the form of a game.
3. Mental well-being and autonomy	Stimulate client to do as much as possible herself. Regularly ask client what she wants and how she would like it.
4. Physical well-being and health	Client can wash herself. When waking up client, offer her a warm wash cloth. Client selects her clothes herself and showers on Monday. When taking a shower, escort client to the shower and regulate the temperature of the shower. Leave a towel on the floor to prevent slipping. Then, client can shower independently. Client goes to the toilet independently and walks independently without any help.

##### Fidelity

The way in which MRC was applied differed from its core principles, as staff reported difficulties remembering all the information gathered from the training sessions. Improved cooperation with other disciplines is observed, and MRC is regularly discussed during staff meetings, yet it was not recorded how often. The staff did not consistently report their actions with the participants in the daily care reports of the patients.

##### Exposure

Movement has become a part of the daily care, however understaffing makes optimal implementation of MRC challenging. Members of the focus group were able to stimulate independence during meals, bedtime, and in ADL, but reported more difficulties in stimulating activities such as walking.

##### Satisfaction

In general, the staff were enthusiastic about MRC. The questionnaires indicate that 78.6% of the nursing staff experience benefits from MRC, compared to 22% of family caregivers. Although only a small group of family caregivers experience benefits from MRC, 89.5% were willing to continue with MRC.

##### Barriers

Understaffing and limited time available for personalized care and stimulation were the most prominent barriers to implementation. Additionally, doubts were expressed about the point at which stimulation becomes a burden for the participants, as some participants decline quickly. The focus group recommended additional training sessions, more consultations with other disciplines, and more time for implementation. The staff did not consistently report their actions with the participants in their daily care reports. Overall, family caregivers and volunteers spent less time stimulating a physically active lifestyle compared to nursing staff.

## Discussion

In the present study, MRC was hypothesized to result in a maintenance, or a slower decline of ADL independence and QoL in NH residents with moderate to severe dementia. Although our study provided no clear evidence for the effectiveness of MRC in improving independence in ADL or QoL, we did find a significant group-by-time interaction for a subscale of the Qualidem. Specifically, after 12 months, residents who received MRC showed a higher positive self-image compared to the control group. A higher positive self-image could indicate that the MRC group verbally expressed less feelings of incapability, less desire for help, and less indications of worthlessness and being a burden to others. No additional benefits of MRC were demonstrated regarding other QoL aspects, which is in accordance with previous studies [12]. Differences in positive self-image between the groups only became apparent after 12 months, which may be explained by the time needed to effectuate environmental changes in the nursing home [5]. It could also indicate the time needed for MRC to have an effect on the patients. However, there were no overall trends of improvements after 12 months.

There are several explanations for the lack of benefits of MRC on QoL. First, it may be justified by the lack of improvements in independent functioning in ADL, as functional independence is an important predictor for QoL [4]. Second, it is possible that QoL is affected by other factors not accounted for in the present study, such as social functioning, social support, and educational level [33]. Nonetheless, the results are indicative of a continued trend in maintenance in positive self-image after a longer intervention period. This finding is relevant as elderly with dementia often struggle with feelings of worthlessness [34]. The role of the environment in encouraging the residents’ self-value has previously been emphasized, as a decreased positive self-image is found to be associated with increased feelings of social rejections [34].

The lack of improvements in ADL are inconsistent with a recent review comparing 15 interventions of which 60% found improvements in ADL [12]. Other similar studies did not show improvements in ADL [18, 19]. The lack of benefits of the intervention may be related to the treatment fidelity. The process evaluations show that even though all 37 participants of the intervention group were reached, some parts of the protocol were not implemented as intended. For example, the nursing staff tended to implement MRC in their own way, as the information gathered from the training session is forgotten with time. Additionally, the family caregivers, activity leaders, and volunteers were not actively stimulating independence and physical activity. The process evaluations also show that nursing staff reported successful implementation of MRC during ADLs such as morning care, meals, and self-care, but delivered less MRC throughout the day with, for example, walking, due to understaffing. Nursing staff were also hesitant about residents’ remaining capabilities, and we anticipate that their fear for resident falls or injury may have prevented them from implementing MRC in all aspects of care, even though medical staff tried assisting them in this aspect. Fear of falling and injury is a common barrier to implementation [35], even though the implementation of function focused care does not increase fall risk [12]. Therefore, the amount of MRC delivered was less than intended, and intensity of the intervention is strongly related to beneficial outcomes [15, 30]. Perhaps incorporating motivational techniques and more staff guidance and information during the implementation would increase treatment fidelity [20].

The process evaluations indicate that the nursing staff were prepared to deliver MRC, and the majority experienced benefits of MRC. The fact that the nursing staff experienced considerably more benefits of MRC compared to family caregivers may be explained by the fact that the nursing staff were involved daily with the care of the participants, enabling them to observe the participant in all routines and activities.

### Strengths and limitations

The strengths of this study include a long intervention period, and outcome measures specifically designed for elderly with dementia. In contrast to previous studies, using the Qualidem enabled us to focus not only on social and emotional domains, but also on care relationship and coping with the NH environment [27]. The strength of MRC is the multidisciplinary and person-centered approach to NH dementia care.

The current study has some limitations. First, the quasi-experimental design is more susceptible to bias than a randomized controlled trial [36]. However, by adjusting for confounders, we partly controlled for potential baseline differences that existed between groups. In addition, there was a limited power as a result of the relatively small sample size. Even though we managed to include only a small sample, these findings are still of value to the limited number of studies that investigate the effect of ADL interventions in elderly with moderate to severe dementia. Still, results should be interpreted with caution. Third, it was difficult to monitor the intensity of the intervention, as the intervention was individually-based and appealed to general health care facets and activities. However, using elaborate process evaluations was highly valuable as it provided insight into the fidelity of the treatment. Last, it was not possible to blind the nursing staff who filled in the questionnaires. This may have caused some bias in resident outcomes due to the subjective nature [37].

## Conclusion

In the current study, the benefits of MRC are limited to a higher positive self-image for the MRC group compared to care as usual after a 12-month intervention period. No improvements in total QoL or ADL were found. Nevertheless, it is important to continue research in this area, as it has the potential to enhance NH dementia care. Studying interventions that reduce ADL dependency will benefit the nursing staff and possibly increase QoL of residents. Understaffing and limited time are important barriers that should be taken into consideration when implementing MRC interventions. Publishing non-significant results is essential in order to avoid exaggeration of benefits of interventions, and contributes to our knowledge on dementia health care. Further large scale studies are required to draw more profound conclusions regarding the impact of MRC on independence in ADL and QoL.

## Abbreviations

ADL: Activity of Daily Living
MMSE: Mini-Mental State Examination
MRC: Movement-oriented Restorative Care
NH: Nursing home
QoL: Quality of Life
